# Programmed cell death in gouty nephropathy: molecular mechanisms and therapeutic implications

**DOI:** 10.3389/fimmu.2026.1817722

**Published:** 2026-05-25

**Authors:** Shengyi Zhao, Bingbing Chen, He Qian, Rong Wang, Sanjin Zeng, Heguo Yan, Jian Zhang, Zhaohu Xie, Zhaofu Li

**Affiliations:** 1 School of Basic Medical Sciences, Yunnan University of Chinese Medicine, Kunming, China; 2 First Clinical Medical College, Yunnan University of Chinese Medicine, Kunming, China

**Keywords:** autophagy, ferroptosis, gouty nephropathy, hyperuricemia, monosodium urate crystals, necroptosis, NLRP3 inflammasome, programmed cell death

## Abstract

Gouty nephropathy (GN) is a chronic kidney disorder driven by persistent hyperuricemia; however, its pathogenesis remains under-investigated. Emerging evidence highlights that programmed cell death (PCD) plays a central role in GN, linking disordered urate metabolism to inflammation and renal functional decline. In GN, multiple PCD modalities, including apoptosis, necroptosis, pyroptosis, ferroptosis, and autophagy, are aberrantly activated. These death pathways contribute to tubular epithelial cell loss, interstitial inflammation, and fibrosis, forming an interactive pathological network rather than isolated phenomena. Apoptosis quietly depletes nephrons; necroptosis and pyroptosis mediate membranolytic cell rupture and sterile inflammation; ferroptosis inflicts iron-dependent lipid peroxidation injury; and autophagy, a double-edged sword, can both mitigate and exacerbate damage through its crosstalk with other PCD pathways. Recognizing the PCD network as a core driver of GN opens new avenues for therapy beyond conventional urate-lowering. Targeting key nodes of cell death holds promise to interrupt the self-perpetuating cycle in GN, for example: restoring mitochondrial redox balance to limit apoptosis, inhibiting the RIPK3/MLKL necroptosis cascade, suppressing NLRP3 inflammasome-mediated pyroptosis, modulating iron homeostasis to prevent ferroptosis, or fine-tuning autophagy. In sum, deciphering the molecular interplay of PCD in gouty nephropathy not only deepens our understanding of its pathology but also reveals novel therapeutic opportunities to protect the kidney beyond simply lowering uric acid levels.

## Highlights

- Multiple forms of programmed cell death are simultaneously activated in gouty nephropathy and drive tubular injury and inflammation.
- Hyperuricemia and monosodium urate crystals converge on shared upstream stressors, forming an interactive network of apoptosis, necroptosis, pyroptosis, ferroptosis, and autophagy.
- Targeting the programmed cell death network offers therapeutic opportunities beyond conventional urate-lowering strategies.

## 1 Introduction

Gouty nephropathy (GN), also known as hyperuricemic nephropathy, is characterized by persistently elevated uric acid levels leading to urate crystal deposition in the kidneys (1, 2). Clinically, GN often presents insidiously, contributing to a hidden burden of chronic kidney disease (CKD) in patients with gout (3). Its manifestations include intrarenal monosodium urate (MSU) crystal deposits, chronic urate stone formation, tubulointerstitial nephritis, and progressive fibrosis (4). Persistent hyperuricemia is prevalent among patients with gout and is increasingly recognized as an independent risk factor for CKD progression (5). The traditional understanding posited that renal impairment in gout results mainly from mechanical crystal obstruction and uric acid precipitation in tubules. However, this view is incomplete; many hyperuricemic patients incur renal damage even before overt crystal deposition occurs, indicating that soluble urate can be pathogenic via more subtle mechanisms (6). Indeed, mounting evidence suggests that gouty kidney injury is not merely a passive consequence of crystal accumulation but is actively driven by inflammatory and molecular events initiated by urate (3).

**Figure 1:**
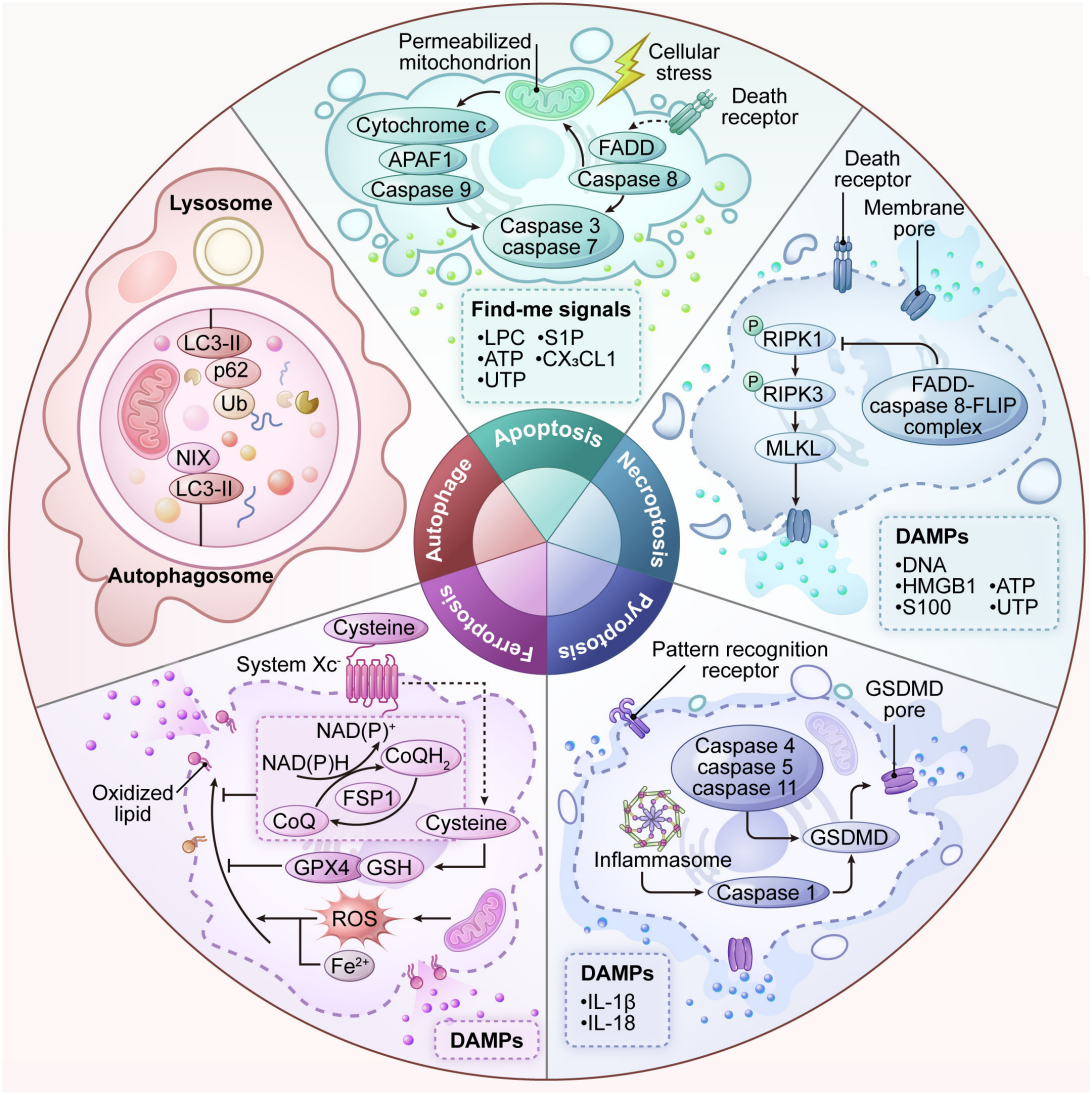
Overview of the five major PCD pathways in GN. Apoptosis mediates “silent” tubular cell loss through ROS-induced mitochondrial damage and Bax/Bcl-2–caspase-9/3 signaling. Necroptosis, driven by the RIPK1–RIPK3–MLKL axis under inflammatory conditions such as TNF-α stimulation, leads to membrane rupture and DAMP release. Pyroptosis links urate crystal sensing to inflammation via NLRP3 inflammasome activation, caspase-1–dependent gasdermin D cleavage, and IL-1β/IL-18 releases. Ferroptosis is triggered by iron-dependent lipid peroxidation and impaired GPX4/xCT antioxidant defenses, promoting tubular injury and fibrosis. Autophagy acts as a context-dependent regulator that can limit oxidative stress and inflammasome activity, but when dysregulated may promote lysosomal damage and inflammatory cell death, thereby modulating the overall PCD network. APAF1, apoptotic protease activating factor-1; FADD, Fas-associated death domain protein; LPC, lysophosphatidylcholine; S1P, sphingosine 1-phosphate; ATP, adenosine triphosphate; CX_3_CL1, C-X3-C motif chemokine ligand 1; UTP, uridine triphosphate; RIPK, receptor-interacting protein kinase; MLKL, mixed-lineage kinase domain-like protein; HMGB1, high mobility group box 1; GSDMD, gasdermin D; GPX4, glutathione peroxidase 4; GSH, glutathione; LC3-II, microtubule-associated protein 1 light chain 3 II; Ub, ubiquitin; NIX, NIP3-like protein X.

A unifying mechanism that connects disturbances in urate metabolism, innate immune activation, and renal functional loss in GN is programmed cell death (PCD). PCD encompasses a spectrum of regulated cellular self-destruction pathways, which are distinct from accidental necrosis and include apoptosis, necroptosis, pyroptosis, ferroptosis, and autophagy-dependent cell death (7) (**Figure 1**; **Table 1**). These pathways are tightly controlled by specific signaling molecules and can be triggered by the hyperuricemic milieu (8). In GN, the excessive urate load and resultant crystal formation subject renal cells, particularly tubular epithelial cells, to continuous stress, which in turn activates PCD programs. This activation leads to cell loss and the release of pro-inflammatory signals (9). Unlike the simplistic model of injury in which urate crystals mechanically damage cells, PCD represents an active and gene-regulated process. In this process, cells execute their death in response to urate-induced signals, thereby linking metabolic disturbance to immune-driven tissue damage.

**Table 1:** The major PCD pathways involved in GN.

PCD modality	Core molecules and pathways	Major triggers	Predominant cell types	Pathological relevance
Apoptosis	Bax/Bcl-2, caspase-9/3, mitochondrial pathway	Intracellular urate, ROS, mitochondrial damage	Tubular epithelial cells	Silent nephron loss, tubular atrophy
Necroptosis	RIPK1–RIPK3–MLKL	MSU crystals, TNF-α, inflammatory milieu	Tubular cells, macrophages	Membrane rupture, DAMP release, inflammation
Pyroptosis	NLRP3, caspase-1, GSDMD, IL-1β	MSU crystals, lysosomal rupture, ROS	Tubular and immune cells	Sterile inflammation amplification
Ferroptosis	Iron metabolism, GPX4, xCT, lipid ROS	Iron overload, impaired antioxidant defense	Tubular epithelial cells	Lipid peroxidation, fibrosis
Autophagy	LC3, Beclin-1, p62, lysosome	Hyperuricemia, metabolic stress	Tubular cells, macrophages	Context-dependent protective or pathogenic role

Recent studies underscore that inflammation, oxidative stress, and regulated cell death mediated by uric acid and urate crystals are intertwined in GN pathogenesis (4). Renal tubular cells may undergo apoptosis as a result of oxidative mitochondrial damage (10). They can also succumb to necroptosis and pyroptosis when exposed to crystal-induced inflammasome and death-receptor signaling, and may even experience ferroptotic death triggered by lipid peroxidation (9). Autophagy, a cellular stress response, is activated by hyperuricemia. While this process can be renoprotective by clearing harmful cellular substrates, its dysregulation may paradoxically contribute to tissue injury. These PCD processes do not act in isolation; instead, they form an interconnected network characterized by crosstalk and positive feedback loops (9). For example, necroptotic cell lysis releases damage-associated molecular patterns (DAMPs), which in turn stimulate inflammasomes and promote pyroptosis (5, 9). Similarly, mitochondrial oxidative stress associated with apoptosis can exacerbate ferroptosis or inflammasome activation, whereas autophagic failure can intensify other death pathways. Therefore, GN can be understood as a chronic condition in which elevated urate levels and crystal deposition initiate a self-amplifying PCD-centric injury cycle.

In this review, we present a comprehensive overview of the molecular mechanisms by which PCD contributes to GN and discuss how these pathways intersect. We first outline the pathophysiological setting of GN, specifically explaining how sustained hyperuricemia and MSU crystal deposition create a pro-death microenvironment in the kidney. We then detail each major PCD modality implicated in GN, which includes apoptosis, necroptosis, pyroptosis, ferroptosis, and autophagy, and we highlight the experimental evidence for their activation and roles in urate-induced renal injury. Next, we examine how these death pathways form an interactive network that shares upstream triggers and within which they mutually influence each other to drive inflammation and tissue damage. Finally, we discuss the therapeutic implications of targeting the PCD network in GN, representing an emerging paradigm that could augment traditional urate-lowering therapy by directly protecting renal cells from demise. By illuminating the central role of PCD in GN, we aim to identify novel intervention strategies and stimulate further research into this under-recognized aspect of gout’s end-organ damage (11).

## 2 Pathophysiology of GN: hyperuricemia and crystal deposition

**Figure 2:**
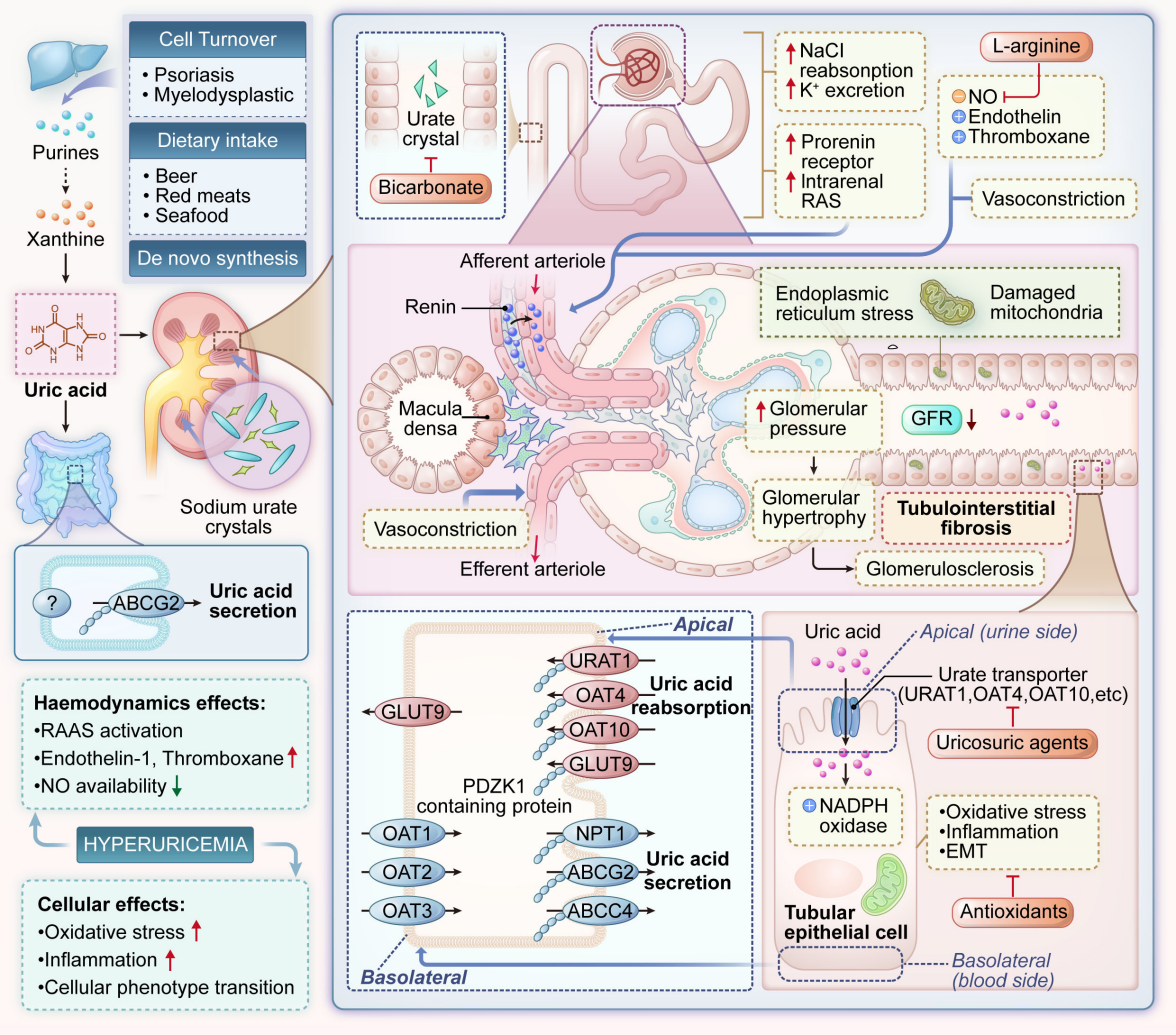
The generation, transport, and excretion of uric acid, as well as its pathogenic roles in GN. Uric acid is produced from purine metabolism mainly through xanthine oxidase activity in the liver and intestine, and is eliminated predominantly by the kidney. Renal urate handling involves a coordinated network of urate transporters, including URAT1 and GLUT9 mediating tubular reabsorption, and OAT1/3, ABCG2, and other transporters facilitating urate secretion. Elevated uric acid exerts deleterious effects through both crystal-dependent and crystal-independent mechanisms. Monosodium urate (MSU) crystal deposition causes tubular obstruction and activates innate immune responses, whereas soluble uric acid induces renal injury by promoting oxidative stress, mitochondrial dysfunction, endothelial impairment, and intrarenal hemodynamic alterations. Together, these metabolic, hemodynamic, and cellular effects converge to drive tubular damage, sterile inflammation, and progressive renal dysfunction in GN. ABCG2, ATP-binding cassette transporter G2; RAAS, renin-angiotensin-aldosterone system; NO, nitric oxide; GFR, glomerular filtration rate; URAT1, urate transporter 1; OAT, organic anion transporter; GLUT, glucose transporter; NPT, sodium/phosphate cotransporter.

Chronic hyperuricemia is the central driving force in the initiation and progression of GN (12). When serum urate concentrations chronically exceed the solubility threshold (approximately 6.8 mg/dL), MSU crystals tend to form and deposit within the kidney (13). The typical deposition sites are the renal tubule lumens, especially in the collecting ducts and distal tubules where urine becomes most concentrated, as well as within the medullary interstitium (4). Over time, these crystals can aggregate into microtophi or stones. One direct consequence involves mechanical obstruction and epithelial injury, as MSU crystals may plug tubular lumens, causing intratubular pressure buildup and local ischemic injury (14). The sharp edges of these crystals can also puncture or erode tubular epithelial cells (14). Indeed, histopathological examination of gouty kidneys reveals crystal deposits surrounded by foreign-body giant cells and fibrotic tissue, indicating a chronic reaction to these persistent irritants (15). Notably, in the classic presentation of “urate nephropathy, ” early pathological lesions consist of crystal deposition in the collecting ducts and renal pelvis, often forming urate stones, accompanied by a chronic interstitial nephritis with fibrotic changes radiating from these sites (16) (**Figure 2**).

Beyond physical blockage, MSU crystals incite a potent innate immune response in the renal microenvironment (17). Renal resident macrophages and dendritic cells can endocytose urate crystals, which triggers their activation. MSU crystals are recognized by pattern recognition receptors. They can bind to cell surface receptors such as CD14 and toll−like receptor (TLR)-2/4 (18, 19). Additionally, once phagocytosed, these crystals can cause lysosomal damage, leading to the release of cathepsin proteases into the cytosol (20, 21). This sequence constitutes a well-known trigger for the NOD-, LRR- and pyrin domain-containing protein 3 (NLRP3) inflammasome (22, 23). In macrophages, internalized crystals lead to lysosome rupture and the subsequent leakage of cathepsin B, an event that directly activates NLRP3 (20). The activated NLRP3 inflammasome in turn recruits caspase-1, which then cleaves pro-IL-1β and pro-IL-18 into their active inflammatory cytokine forms. These cytokines, particularly IL-1β, are subsequently secreted and initiate a cascade of inflammation and further cell recruitment (6, 15). This sterile inflammatory process, often termed necroinflammation, is central to gout’s pathology (24). Even though no infection is present, the crystals act as endogenous danger signals that provoke a vigorous immune attack, ultimately damaging local tissue.

Hyperuricemia is pathogenic even at a pre-crystalline stage. Soluble uric acid can enter renal tubular cells through specific transporters such as urate transporter 1 (URAT1, a.k.a., SLC22A12) on the apical membrane of proximal tubules (25). Once inside the cell, urate perturbs cellular homeostasis through multiple mechanisms (26). One key effect is the induction of oxidative stress. As a pro-oxidant, intracellular uric acid stimulates the production of reactive oxygen species (ROS), for example by activating NADPH oxidases (27). Excess ROS leads to mitochondrial dysfunction, characterized by a collapse of mitochondrial membrane potential and the release of pro-apoptotic factors, and can also damage DNA, proteins, and lipids in renal cells (28). Additionally, urate can consume nitric oxide and impair endothelial function, which reduces medullary blood flow and exacerbates ischemic stress in the kidney (6). Hyperuricemia is further linked to activation of the renin–angiotensin system and pro-inflammatory signaling in the kidney, thereby compounding the overall injury (29).

Multiple pathological changes in the hyperuricemic kidney create a confluence of factors conducive to activating PCD pathways. High levels of ROS not only directly induce cell death, such as through apoptosis or ferroptosis, but also function as signaling molecules that amplify inflammatory responses (30). For instance, urate-induced ROS can activate the transcription factor NF-κB (31), which in turn primes the NLRP3 inflammasome by upregulating the expression of both pro–IL-1β and NLRP3 itself (32). Mitochondrial damage leads to the release of mitochondrial components, including DNA and cardiolipin, into the cytosol (33). These molecules serve as endogenous danger signals (i.e., DAMPs) that can activate inflammasomes or the cGAS-STING signaling pathway. Lysosomal membrane instability resulting from crystal overload causes the release of cathepsins, as previously noted. This not only lowers the threshold for inflammasome activation but can also initiate cell death through lysosome-dependent mechanisms. Furthermore, urate disrupts ion homeostasis. MSU crystals, for example, can induce potassium efflux from cells, which is a well-established trigger for NLRP3 activation (34), and can perturb intracellular calcium signaling, a common feature in cellular demise (35). Additionally, even iron homeostasis may be compromised, with evidence suggesting that hyperuricemia can dysregulate iron transport proteins such as ferroportin-1 (36, 37). This dysregulation leads to iron accumulation in renal tubules (38). Free iron then catalyzes the generation of additional ROS via Fenton reactions, linking back to oxidative injury and predisposing cells to ferroptosis.

Sustained high urate levels and crystal deposition thus orchestrate a transition in the kidney—from a state of metabolic imbalance to a chronic disease state defined by the interplay of cell death and inflammation (16). In this pathological landscape, renal tubular epithelial cells become the primary targets, enduring a multifaceted assault: internalized urate drives oxidative stress and apoptosis, external crystal irritation induces membrane damage and necroptosis, and inflammatory cytokines from immune cells can trigger apoptosis or pyroptosis (39). The resulting attrition of tubular cells through persistent PCD leads to nephron loss and tubular atrophy (16). Moreover, debris from dead cells, such as ATP, high-mobility group box 1 (HMGB1), and DNA fragments, functions as DAMPs that further amplify immune activation and perpetuate an inflammatory cycle (40). This is accompanied by the activation of interstitial fibroblasts and infiltration of monocytes, processes that collectively drive fibrosis, the hallmark of progressive CKD (41). Viewed through this lens, GN emerges as a form of CKD initiated by hyperuricemia and crystals but fundamentally sustained by a dynamic network of PCD pathways and sterile inflammation. This mechanistic framework sets the stage for a detailed examination of each individual PCD modality and its distinct contribution to renal pathology in gout.

## 3 Major PCD pathways in GN

### 3.1 Apoptosis: high uric acid–induced “silent” death of tubular cells

Apoptosis is a form of PCD characterized by programmed cellular self-destruction that does not provoke inflammation (42, 43). In GN, chronic exposure to elevated uric acid levels can induce apoptosis in renal tubular cells, leading to a covert loss of nephrons over time (27). Mechanistically, urate-triggered apoptosis in kidney cells primarily follows the intrinsic, or mitochondrial, pathway (27). Experimental studies using human proximal tubule cell lines (such as HK-2) have demonstrated that uric acid exposure reduces cell viability and increases the apoptotic cell population in a dose-dependent manner (27). For example, these studies reported up to a seven-fold increase in TUNEL-positive cells (27). Uric acid enters tubular cells via the URAT1 transporter (27). Once inside, it stimulates excessive production of ROS, partly through upregulation of NADPH oxidase NOX4 (27, 44). The surge in ROS inflicts mitochondrial injury, as evidenced by the loss of mitochondrial membrane potential and the release of cytochrome c (27). These events collectively activate the pro-apoptotic protein Bax while simultaneously suppressing anti-apoptotic factors such as Bcl-2 (4, 27). The resulting cascade then activates caspase-9, the initiator caspase of the intrinsic pathway. Activated caspase-9 subsequently cleaves and activates the executioner caspase-3, which orchestrates the systematic dismantling of cellular components and mediates DNA fragmentation. Notably, *in vitro* studies have shown that inhibiting caspase-9—but not caspase-8—significantly attenuates urate-induced apoptosis (27). This finding confirms the dominant role of the mitochondrial pathway in this specific pathological context.

Urate-induced apoptosis can be thought of as a “silent” form of cell death because apoptotic cells neatly package themselves into apoptotic bodies that are cleared by phagocytes without releasing pro-inflammatory content (45). However, its impact on kidney structure and function is insidious. Continuous apoptotic attrition of tubular epithelial cells contributes to tubular atrophy and loss of functional nephron units in hyperuricemic nephropathy (45). Morphologically, renal biopsies from hyperuricemic models show shrunken, atrophic tubules and thinning of the epithelium, consistent with chronic apoptotic cell loss (46). Apoptosis in tubular cells is also linked to the development of interstitial fibrosis (16). As epithelial cells undergo apoptosis, they initiate pro-fibrotic changes within the surrounding interstitium, a process sometimes described as “atrophic tubule signaling.” This occurs through mechanisms including the release of TGF-β from dying cells and the breakdown of epithelial integrity, which in turn facilitates inflammatory cell infiltration and a pro-fibrotic microenvironment.

Multiple signaling pathways converge on mitochondria to mediate urate-induced apoptosis (27). Beyond the role of ROS, elevated intracellular uric acid can activate the p53 pathway (47, 48). Specifically, urate has been shown to stabilize the p53 protein, leading to its transcriptional upregulation of Bax and other pro-apoptotic genes (27). Furthermore, extracellular urate crystals can engage death receptors such as Fas or tumor necrosis factor (TNF) receptor on the surface of tubular cells. This interaction is likely facilitated by inflammatory mediators like FasL or TNF-α released from neighboring immune cells (49). Activation of these death receptors initiates the extrinsic apoptotic pathway through the adaptor protein FADD (Fas-associated death domain) and caspase-8. Evidence suggests that both intrinsic and extrinsic pathways operate in GN. For instance, one study demonstrated elevated TNF-α levels in hyperuricemic kidneys and observed that blocking TNF-α signaling reduced tubular cell apoptosis. Nevertheless, activation of the intrinsic pathway via oxidative stress appears to be predominant, as apoptosis in urate-stressed tubular cells was significantly attenuated by ROS scavengers and pharmacological inhibition of NOX4 (27). Importantly, pharmacological agents that inhibit urate uptake—such as probenecid or losartan, which target the URAT1 transporter—markedly reduce tubular apoptosis (27). These findings underscore that intracellular urate accumulation serves as the critical trigger for apoptosis. Merely elevated extracellular urate is insufficient; the internalization of uric acid and the subsequent intracellular perturbations are necessary to initiate the apoptotic program.

### 3.2 Necroptosis: crystal and inflammation-driven membrane-rupture cell death

Necroptosis is a form of regulated necrosis that follows a programmed pathway, resulting in cell membrane rupture and the release of intracellular contents, which in turn induces inflammation (50). This process is primarily executed through the sequential activation of receptor-interacting serine/threonine protein kinase 1 (RIPK1), RIPK3, and the pseudokinase mixed-lineage kinase domain-like protein (MLKL) (51). In GN, necroptosis has been recognized as an important contributor to tubular injury, particularly in the context of MSU crystal deposition and inflammatory cytokine activity (15). Unlike apoptosis, which leads to orderly cell death, necroptosis culminates in cell lysis and exhibits a necrotic morphology, rendering it highly pro-inflammatory and capable of amplifying tissue damage.

MSU crystals can directly induce necroptotic cell death in various cell types, including renal tubular epithelial cells and infiltrating immune cells (15). A landmark study demonstrated that urate crystals, along with other pathogenic crystals such as oxalate, trigger a form of caspase-independent cell death that can be prevented by necroptosis inhibitors (15). Specifically, MSU crystal-induced cell death was shown to be blocked by Necrostatin-1, a selective inhibitor of RIPK1, indicating the involvement of the RIPK1-RIPK3 pathway (15). Furthermore, genetic silencing or knockout of RIPK3 or MLKL protected cells from MSU crystal cytotoxicity (15). These findings indicate that crystal-induced cell death is not merely a consequence of physical damage but involves the active engagement of the necroptotic machinery. The underlying mechanism may involve crystal-induced perturbations of the plasma membrane or endosomal compartments, which signal through RIPK1, or may be mediated by secondary signals such as TNF-α released from crystal-stimulated macrophages (15).

Inflammatory cytokines present in GN can synergize with crystals to promote necroptosis (52). TNF-α, which is elevated in hyperuricemic kidneys (5), serves as a potent inducer of necroptosis under conditions where caspase-8 activity is inhibited or overwhelmed. In GN, TNF-α likely binds to TNFR1 on tubular epithelial cells and initiates the assembly of a signaling complex containing RIPK1. Under normal conditions, caspase-8 would cleave RIPK1/RIPK3, thereby inhibiting necroptosis. However, within the highly inflammatory microenvironment of GN, which may involve factors such as XIAP (X-linked inhibitor of apoptosis) depletion that contribute to subtle caspase inhibition, RIPK1 instead forms a complex known as the necrosome with RIPK3 (53, 54). Subsequently, RIPK3 phosphorylates MLKL, prompting its oligomerization and translocation to the plasma membrane, where it forms disruptive pores. This cascade culminates in cell membrane rupture, representing a form of programmed cellular disintegration. The process results in the release of DAMPs such as HMGB1, ATP, and nuclear proteins into the interstitial space. These DAMPs further perpetuate inflammation, a cycle often referred to as necroinflammation (52). This involves the recruitment of neutrophils and monocytes, along with the activation of fibroblasts, thereby exacerbating tissue injury and contributing to disease progression.

Animal models provide strong evidence supporting the role of necroptosis in hyperuricemic kidney injury. In a mouse model of hyperuricemia induced by oxonic acid, renal tissue exhibited significant upregulation of RIPK3 (5). Strikingly, genetic deletion of RIPK3 not only reduced renal cell death but also improved overall renal function. Despite comparable levels of hyperuricemia, RIPK3-knockout mice demonstrated lower serum creatinine and less severe histological damage. These animals also showed reduced oxidative stress markers, such as lower malondialdehyde and ROS levels, along with decreased expression of inflammatory cytokines in the kidneys (5). Furthermore, the absence of RIPK3 led to suppressed activation of TLR4/NF-κB signaling and diminished NLRP3 inflammasome activity in hyperuricemic mice (5). This finding is critical as it suggests that necroptosis, mediated by RIPK3, may function upstream of or in close interaction with inflammasome activation and cytokine release. Indeed, RIPK3 deficiency was associated with lower levels of IL-1β, TNF-α, and IL-6 in both systemic circulation and renal tissue (5). Additionally, markers of apoptosis, including cleaved caspase-3/-8, and PARP, were reduced in RIPK3-knockout hyperuricemic kidneys, suggesting potential crosstalk between necroptosis and apoptotic pathways, possibly via RIPK1-FADD-caspase-8 signaling complexes (5). Collectively, these results position necroptosis as a central mediator of cell death and inflammation in urate-induced nephropathy.

Clinically, although direct evidence in human GN is limited, insights can be drawn from related pathological conditions. For instance, renal biopsies from patients with acute crystal nephropathy, such as oxalate nephropathy, have shown tubular epithelial cells positive for phosphorylated MLKL, a hallmark of necroptosis (15). It is plausible that in chronic GN, ongoing sub-lethal crystal deposition induces low-grade necroptotic death, which cumulatively contributes to renal damage over time (15). Necroptosis may be particularly pronounced in anatomical segments where crystals frequently lodge, such as the medullary collecting ducts. Moreover, necroptosis occurring in infiltrating leukocytes, including macrophages, can result in the release of proteases and ROS that further injure surrounding tubular structures.

### 3.3 Pyroptosis: an inflammatory cell death linking crystals to immune response

Pyroptosis is a highly inflammatory form of PCD, typically triggered by inflammasome activation (55). It is characterized by cellular swelling, the formation of lytic pores in the plasma membrane, and the release of potent pro-inflammatory cytokines such as IL-1β and IL-18. This process is mediated by members of the gasdermin family of pore-forming proteins, particularly gasdermin D (GSDMD), which is cleaved and activated by caspases. In the canonical pathway, caspase-1 performs this cleavage, while in non−canonical pathways, caspases−4, −5, or −11 are involved. In the context of GN, pyroptosis serves as a crucial mechanistic link that directly connects the deposition of MSU crystals with the intense inflammatory response observed in renal tissue (56).

The cornerstone of pyroptosis in GN is the NLRP3 inflammasome. As described earlier, MSU crystals are one of the most potent activators of the NLRP3 inflammasome (57). Within macrophages and possibly within resident renal cells such as tubular epithelial cells, urate crystals promote NLRP3 assembly, typically through triggers that include lysosomal damage and potassium efflux. Once assembled, the NLRP3 inflammasome recruits and activates caspase-1. Active caspase-1 then performs two key functions: it cleaves the precursor cytokines pro-IL-1β and pro-IL-18 into their active (58), secreted forms, and simultaneously cleaves the pore-forming protein GSDMD. Caspase-1−mediated cleavage liberates the N−terminal fragment of GSDMD, which oligomerizes and inserts into the plasma membrane to form pores approximately 10–15 nm in diameter (59). These pores facilitate the efflux of mature IL-1β and IL-18, along with other intracellular constituents such as ions and lactate dehydrogenase. The consequent loss of osmotic balance leads to rapid cellular swelling and eventual plasma membrane rupture. In essence, the pyroptotic cell undergoes a form of programmed lytic death, releasing a burst of inflammatory mediators into its surroundings. The secreted IL-1β and IL-18 then act on neighboring cells to induce the production of chemokines, promote febrile responses, and amplify local inflammation, thereby recruiting neutrophils and monocytes into renal tissue.

Both immune and resident renal cells can undergo pyroptosis in the context of GN. Macrophages that ingest MSU crystals are well-known to undergo pyroptosis; this is in fact a protective mechanism to some extent, as it sacrifices the macrophage but sends out alarm signals such as IL-1β to recruit more immune cells to deal with the threat (60, 61). In a mouse model of gout-like inflammation, knockout of key pyroptosis components (e.g., NLRP3, ASC, or caspase-1) led to reduced neutrophil infiltration in response to MSU, and macrophages from these mice released far less IL-1β upon MSU stimulation (60). This underscores that MSU-induced IL-1β release *in vivo* largely depends on pyroptosis. In the kidney, tubular epithelial cells themselves can also form inflammasomes and undergo pyroptosis (62). Studies have found that exposing proximal tubular cells to high urate or MSU crystals activates NLRP3 and caspase-1 within these cells, resulting in GSDMD cleavage and IL-1β secretion (20). Pyroptotic death of tubular cells would directly compromise the nephron and simultaneously propagate inflammation *in situ* (63). Additionally, other renal cell types, such as endothelial cells, may contribute to this process. For example, hyperuricemia has been shown to promote endothelial pyroptosis via ROS−NLRP3 pathways in other vascular contexts, such as atherosclerosis (64, 65). A similar mechanism could occur in the renal microvasculature, potentially contributing to peritubular capillary rarefaction and further exacerbating renal injury in GN.

A unique feature of MSU-driven pyroptosis is that MSU crystals simultaneously initiate the transcriptional priming and the inflammasome-activating arms of the NLRP3 pathway (66, 67). Upon phagocytosis, MSU crystals activate NF-κB through TLR2/4 and possibly cytokine receptors, inducing the expression of NLRP3 and pro-IL-1β, while in parallel they destabilize lysosomes to promote NLRP3 inflammasome assembly (68). This coordinated dual input creates a one-two punch that enables rapid and robust IL-1β production. In addition, non-canonical pyroptotic pathways may further amplify this response. Cytosolic LPS, potentially derived from gut microbial translocation in CKD, can activate caspase-11 in mice (caspase-4/5 in humans), leading to GSDMD cleavage and secondary NLRP3 activation (69). Although this pathway is likely secondary in GN, concurrent infections or endotoxemia could exacerbate renal pyroptosis through these non-canonical routes.

Pyroptosis creates a feedback loop in GN’s pathology. IL-1β released from pyroptotic cells not only causes inflammation but can further injure the kidney. IL-1β and IL-18 stimulate tubular cells and interstitial cells to produce chemokines such as monocyte chemoattractant protein−1 (MCP−1) and upregulate adhesion molecules, driving more leukocyte infiltration. IL-1β can also cause endothelial dysfunction and increase vascular permeability (70). Moreover, other DAMPs from pyroptotic cells (e.g., ATP, HMGB1) can activate inflammasomes in neighboring cells or trigger necroptosis and apoptosis. Of particular note is HMGB1, which binds to TLR4 and the receptor for advanced glycation end products (RAGE) on renal cells (71). This engagement amplifies NF−κB signaling and helps sustain a vicious cycle of inflammation and cell death. Supporting this concept, experimental blockade of RAGE in hyperuricemic animal models has been shown to reduce renal inflammation and tissue damage, indicating that DAMP−mediated signaling plays a critical role in propagating injury in GN (72). In this context, NINJ1-mediated plasma membrane rupture may represent an additional terminal mechanism for DAMP release after lytic cell death (73, 74). However, NINJ1 has not yet been directly examined in GN and should therefore be considered a plausible but unvalidated amplifier of MSU-driven sterile inflammation.

### 3.4 Ferroptosis: lipid peroxidation–driven cell death in a high uric acid milieu

Ferroptosis is a distinctive form of PCD caused by iron-dependent oxidative destruction of lipid membranes. It is morphologically and biochemically separate from apoptosis or necrosis, featuring abundant lipid peroxides and a loss of plasma membrane integrity without blebbing or caspase activation (75). In the context of GN, recent evidence suggests that the hyperuricemic environment predisposes renal tubular cells to ferroptosis, thereby contributing to tubular injury and subsequent fibrosis.

Several lines of evidence indicate ferroptosis occurs in hyperuricemic kidneys (36, 76). In a robust 2025 study, uricase-knockout mice—which spontaneously develop hyperuricemia—showed clear signs of ferroptosis in renal tissue (76). These included excessive iron deposition within renal tubules and a pronounced reduction in the expression of key anti−ferroptotic proteins, glutathione peroxidase 4 (GPX4) and the cystine transporter xCT (SLC7A11) (76). GPX4 is an enzyme that detoxifies lipid peroxides, and xCT is a cystine transporter critical for glutathione synthesis; their downregulation indicates impaired defenses against lipid oxidation (36). These hyperuricemic mice developed renal dysfunction, tubulointerstitial inflammation, and fibrosis, all of which are characteristic features of GN (76). When treated with the ferroptosis−specific inhibitor ferrostatin−1, these animals showed significant improvements (76). Fer−1 attenuated tubular damage, reduced inflammatory cell infiltration, and diminished collagen deposition associated with fibrosis (36, 76). Moreover, Fer−1 treatment restored the levels of antioxidant proteins such as GPX4 and normalized lipid peroxidation markers in kidney tissue (36, 76). These results strongly implicate ferroptosis in driving kidney injury under hyperuricemic conditions, and show that blocking ferroptosis can be protective.

*In vitro* experiments complement these findings. Exposure of human proximal tubule cells (HK−2) to MSU crystals induced a ferroptotic phenotype, characterized by increased accumulation of lipid peroxides, depletion of glutathione, and cell death that could be rescued by ferroptosis−specific inhibitors such as liproxstatin−1 (76). Notably, MSU−induced cell death was accompanied by the accumulation of intracellular iron, a key trigger of ferroptosis (76). This effect may be attributed to the disruption of cellular iron homeostasis by MSU and elevated urate. For instance, these factors can impair the function of FPN1, the sole known cellular iron exporter. In GN, if FPN1 expression is downregulated or its activity compromised—potentially due to urate−induced oxidative damage or inflammatory mediators such as hepcidin—iron becomes sequestered within epithelial cells. This dysfunctional iron export leads to intracellular Fe^2+^ overload, which in turn promotes the generation of reactive oxygen species and drives lipid peroxidation, thereby initiating ferroptosis (77).

Hyperuricemia also interacts with central regulators of ferroptosis, particularly the antioxidant transcription factor NRF2 (nuclear factor erythroid 2−related factor 2). Under normal circumstances, NRF2 promotes the expression of genes such as SLC7A11 and GPX4, which are essential for cellular protection against ferroptosis. However, research shows that uric acid can inhibit NRF2 activity and its downstream signaling. This effect may result from urate enhancing the activity of KEAP1, the primary inhibitor of NRF2, or from oxidative modifications that prevent NRF2 from entering the nucleus. By suppressing NRF2 function, hyperuricemia shifts cells toward a pro-ferroptotic state, marked by lower GPX4 levels and a reduced ability to clear lipid peroxides. Supporting this view, uricase-knockout mice show decreased expression of NRF2 target genes in the kidney. In contrast, interventions that activate NRF2, including certain nutraceuticals, have been found to mitigate hyperuricemic kidney injury by increasing GPX4 and glutathione levels (77, 78). For example, pachymic acid, a bioactive triterpenoid component of the medicinal fungus *Poria cocos*, was shown to alleviate fructose-induced hyperuricemic nephropathy through NRF2 activation and subsequent GPX4 upregulation, thereby inhibiting ferroptosis and reducing renal fibrosis (78). Beyond the NRF2–GPX4 axis, the FSP1–CoQ10 pathway provides a GPX4-independent ferroptosis defense by regenerating reduced CoQ10/ubiquinol, which traps lipid peroxyl radicals and limits phospholipid peroxidation (79). Given the lipid peroxidation-rich milieu of hyperuricemic kidneys, this axis may also be relevant to GN, although direct evidence for FSP1–CoQ10 dysregulation in GN is currently lacking (80).

The consequence of ferroptosis in GN is two-fold: direct parenchymal cell loss and the generation of pro-inflammatory and pro-fibrotic signals (81, 82). When tubular cells undergo ferroptosis, their membranes break down due to lipid peroxide damage, which can release DAMPs similarly to necroptosis and pyroptosis (83). A distinctive feature of ferroptosis is the generation of lipid peroxidation byproducts such as 4−hydroxynonenal and malondialdehyde, which can covalently modify proteins to form immunogenic adducts. These adducts are capable of engaging receptors, such as RAGE. The aforementioned study observed elevated RAGE expression in hyperuricemic kidneys, coinciding with increased ferroptosis markers (76). Blocking RAGE signaling improved inflammatory and fibrotic outcomes in those mice, although it did not completely prevent ferroptotic cell death (76). This suggests that ferroptosis releases RAGE ligands (like oxidized molecule adducts or HMGB1) which then act on surrounding cells to drive a fibrotic inflammatory response (83, 84). Fibroblasts, for instance, respond to oxidative stress signals by differentiating into myofibroblasts, depositing collagen (82). Moreover, under hyperuricemic conditions, excess lipid peroxides and iron can directly activate fibrogenic pathways, such as those mediated by TGF−β or NF−κB in interstitial cells, thereby further driving renal fibrosis.

### 3.5 Autophagy: navigating the intersection of various PCD signaling cascades

Autophagy is a cellular catabolic process whereby cells degrade and recycle their own components through lysosomal machinery. It is generally a survival mechanism under stress, removing damaged organelles and proteins. However, dysregulated autophagy can lead to type II programmed cell death, and importantly, autophagy intersects with nearly all other PCD pathways as a major regulator (85). In GN, hyperuricemia and MSU crystals are potent inducers of autophagy in renal cells (86), placing autophagy at a pivotal position – it can either protect against urate-induced damage or, if excessive or impaired, contribute to cell death and inflammation.

Hyperuricemia has been shown to activate autophagy in renal tissues (87). In a rat model of hyperuricemic nephropathy, sustained stimulation with high uric acid led to increased formation of autophagosomes in tubular epithelial cells, as evidenced by electron microscopy and elevated expression of the autophagy marker LC3−II (20). Uric acid appears to induce autophagy primarily through the p53 pathway. In urate−stressed HK−2 cells, for example, accumulation of p53 was observed, which can transcriptionally activate autophagy−related genes such as DRAM1 and thereby initiate the autophagic cascade (20). On one hand, this autophagic response may represent a cellular attempt to counteract the harmful effects of urate. For instance, autophagy can remove damaged mitochondria through mitophagy, thereby reducing ROS production, or it can sequester internalized urate crystals into autophagolysosomes for degradation (88). Supporting a protective role, studies in an osteoblast model demonstrated that phagocytosed MSU microcrystals triggered NLRP3−dependent autophagy, leading to the formation of autophagolysosomes that encapsulated the crystals and limited excessive IL−1β release (11). This suggests that autophagic encapsulation of MSU crystals may serve to isolate them from cytosolic sensors, thereby reducing inflammasome activation—analogous to forming a protective barrier around the hazardous material.

On the other hand, there is compelling evidence that in hyperuricemic nephropathy, autophagy can become maladaptive and actively contribute to injury. A recent study demonstrated that pharmacological inhibition of autophagy—using agents such as 3−methyladenine or small interfering RNA targeting Beclin-1—protected tubular epithelial cells from uric acid−induced damage (20). In urate−treated HK−2 cells, autophagy inhibition prevented the upregulation of fibrogenic markers, including α−smooth muscle actin (α−SMA) and collagen types I and III, which typically accompany chronic urate exposure (20). More strikingly, *in vivo* inhibition of autophagy markedly ameliorated renal injury in hyperuricemic rats, primarily by suppressing inflammasome−mediated pyroptosis (20). The underlying mechanism revealed that excessive autophagic flux under hyperuricemic conditions accelerates autolysosome turnover and ultimately destabilizes lysosomes. As autophagosomes fuse with lysosomes to form autolysosomes and degrade their contents, overactivation of this process can induce lysosomal membrane permeabilization, resulting in the leakage of cathepsin B into the cytosol (20). This released cathepsin B was shown to activate the NLRP3 inflammasome, thus driving caspase−1 activation and pyroptosis (20). In the same study, either silencing cathepsin B or inhibiting autophagy interrupted this cascade, leading to reduced caspase−1 cleavage, decreased release of IL−1β and IL−18, and improved renal function (20). These findings highlight a paradoxical interplay between autophagy and the inflammasome: although autophagy normally serves to degrade inflammasomes as part of a negative−feedback loop, excessive autophagy under sustained urate stress may actually promote inflammasome activation through physical damage to lysosomal integrity.

Autophagy exhibits complex bidirectional interactions with apoptosis and ferroptosis (89). In certain contexts, activation of autophagy can delay or inhibit apoptosis by removing damaged mitochondria and preventing the release of cytochrome c (90). Evidence suggests that the mild autophagic response induced by urate may help eliminate sources of ROS and temporarily protect cells from apoptotic death (20). However, if the cellular stress is excessive or autophagy is insufficient, cells may still undergo apoptosis (91). Conversely, autophagy can itself promote apoptosis when it leads to excessive degradation of essential cellular components, a process sometimes referred to as autophagy−dependent cell death. For example, prolonged or overactive autophagy may deplete critical organelles such as mitochondria or degrade vital proteins, thereby pushing cells beyond their survival threshold (92).

A specific link between autophagy and ferroptosis occurs through ferritinophagy, the selective autophagic degradation of ferritin, which is the primary intracellular iron−storage complex (93, 94). When autophagy is upregulated, it can enhance ferritinophagy via the cargo receptor NCOA4 (nuclear receptor coactivator 4), thereby releasing free iron from stored ferritin (95). In hyperuricemic conditions where autophagy is active, increased ferritinophagy may contribute to the iron overload observed in tubular epithelial cells and heighten their susceptibility to ferroptosis (96). At the same time, autophagy is required to clear damaged lipid peroxides, implying that a baseline level of autophagic activity is protective against ferroptotic cell death (83). The net outcome of autophagy in relation to ferroptosis is thus highly context−dependent and time−sensitive (96). Studies in models of kidney injury indicate that inducing autophagy, for example through AMPK activation, can mitigate ferroptosis by removing oxidized mitochondria and upregulating antioxidant defenses, whereas inhibiting autophagy tends to exacerbate oxidative damage (11). However, as noted previously, beyond a certain threshold, autophagy may shift from a protective mechanism to one that facilitates cell death (97).

Thus, autophagy in GN functions as a double−edged sword. Early or moderate activation of autophagy in response to urate exposure may represent an adaptive, protective response (98). It can help limit tissue damage by clearing inflammasome complexes—cells often employ autophagy to degrade components such as NLRP3—as well as by removing crystals and DAMPs, and by improving cellular metabolism through nutrient recycling (11, 99). In fact, pharmacological enhancement of autophagy using agents such as rapamycin or AMPK activators has been reported in models of gouty arthritis to reduce inflammation, in part through the autophagic degradation of NLRP3 (100). However, under conditions of chronic hyperuricemia, continuous stimulation of autophagy can lead to a state of “autophagic stress, ” in which the system becomes overloaded (20). Inefficient clearance of accumulated autophagosomes or an excessive burden of crystals may then reach a tipping point, triggering inflammatory forms of cell death, as exemplified by the release of cathepsin B and the consequent activation of pyroptotic pathways (20).

In GN, whether to stimulate or inhibit autophagy as a therapeutic strategy presents a nuanced clinical question (92, 101). The optimal approach likely depends on the stage of the disease and the specific cellular context. In patients whose GN is characterized by excessive inflammation and pyroptosis driven by dysregulated autophagy, carefully attenuating autophagic activity may be beneficial (20, 102). Conversely, in cases where autophagy is insufficient and crystals accumulate unabated, enhancing autophagic capacity could offer therapeutic advantages (11, 103). The interplay between autophagy and cellular metabolic status is also crucial. Factors such as the AMP/ATP ratio, mTOR activity, and overall cellular energy balance under hyperuricemic conditions will significantly influence the functional outcome of autophagy (104, 105).

## 4 Networked synergy and cross-talk of PCD pathways

### 4.1 Shared upstream triggers

**Figure 3:**
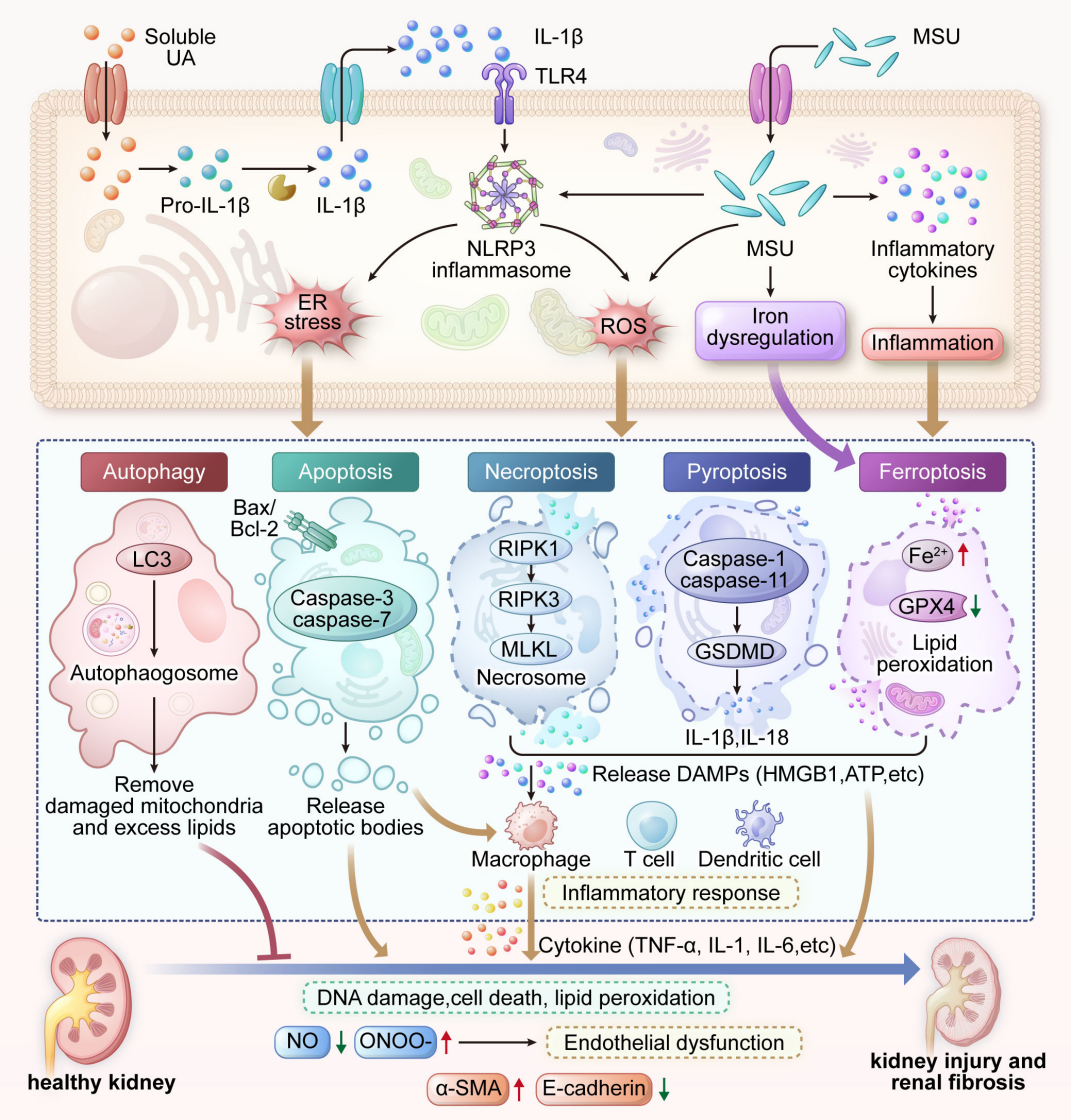
Integrated PCD network driving the progression of GN. Upstream pathological triggers, including soluble uric acid overload, monosodium urate (MSU) crystal deposition, reactive oxygen species (ROS) accumulation, endoplasmic reticulum (ER) stress, iron dysregulation, and pro-inflammatory signaling, converge to activate multiple PCD modalities. These include apoptosis, necroptosis, pyroptosis, ferroptosis, and autophagy-associated cell fate regulation. Rather than acting independently, these pathways interact and amplify each other through shared stress signals and inflammatory mediators, ultimately leading to tubular epithelial cell loss, sterile inflammation, extracellular matrix deposition, and progressive tubulointerstitial fibrosis.

A striking aspect of GN is that the diverse PCD pathways, including apoptosis, necroptosis, pyroptosis, ferroptosis, and autophagy-dependent death, often originate from a common set of upstream stressors (9). Sustained hyperuricemia and urate crystal deposition create an overlapping array of triggers that collectively drive these lethal processes (**Figure 3**).

ROS serve as a central node in this network (106). Excessive ROS is a unifying trigger for apoptosis, pyroptosis, and ferroptosis. High ROS damage mitochondria, leading to apoptosis via the intrinsic pathway (27). Simultaneously, ROS acts as a redox-sensitive second messenger that promotes NLRP3 inflammasome activation and thereby contributes to pyroptosis (32). In ferroptosis, specifically lipid ROS, directly executes cell death by peroxidizing membrane lipids. In the hyperuricemic kidney, urate-induced ROS production, fueled by NADPH oxidase activation and mitochondrial dysfunction (27), primes cells concurrently for intrinsic apoptosis and inflammasome activation. It also establishes the conditions required for ferroptosis when iron and peroxidizable lipids are present.

Lysosomal damage represents another critical trigger. MSU crystals induce lysosomal membrane permeabilization, resulting in the release of cathepsins (107). This event can initiate multiple PCD outcomes (108). Cathepsin B release potently activates the NLRP3 inflammasome, driving pyroptosis (20), while lysosomal rupture can also trigger programmed necrosis or apoptosis through the lysosomal cell death pathway. In that pathway, cathepsins such as cathepsin L cleave cytosolic substrates to initiate apoptotic or necrotic signaling (109). In renal tubular cells of GN, lysosomal destabilization may thus promote both caspase-dependent apoptosis and caspase-1-dependent pyroptosis. Moreover, extensive lysosomal compromise impairs autophagic flux because autophagosomes cannot properly fuse with damaged lysosomes (110). This further promotes inflammasome activation due to the accumulation of undegraded substrates.

Ion dysregulation further contributes to this convergent activation. High urate levels and crystals disrupt ionic homeostasis, particularly of potassium and calcium (111). A decrease in cytosolic K^+^, which can occur through crystal-induced pore formation or ATP-mediated opening of pannexin channels, is a well-established universal trigger of the NLRP3 inflammasome and pyroptosis (112). Conversely, calcium overload can activate calpains and other enzymes that promote necrotic death or apoptosis, the latter via calpain-mediated Bax activation (113). In renal epithelial cells, MSU crystals can elevate intracellular Ca^2+^, likely through membrane damage or release from intracellular stores. This calcium dysregulation exacerbates mitochondrial injury and may activate Ca^2+^-dependent endonucleases that promote apoptosis or phospholipases that cause membrane damage akin to necrosis (114). Such ionic shifts act as broad cellular signals that do not respect the boundaries of a single PCD pathway. Instead, they generate an intracellular environment in which multiple death programs can proceed in parallel.

Inflammatory cytokines and danger signals, released upstream in GN, provide additional common stimuli. Molecules such as TNF-α, IL-1β, and HMGB1 can activate receptors that feed into multiple death pathways (9). TNF-α, for instance, can initiate both apoptosis via TNFR1 and caspase-8 and necroptosis when caspase-8 activity is inhibited within the same cell (115, 116). Although primarily an inflammatory mediator, IL-1β can also induce cell death in certain contexts, such as pyroptosis in macrophages via autocrine signaling or apoptosis through the induction of nitric oxide production. DAMPs like HMGB1 or ATP engage receptors such as TLR4 and P2X7, activating NF-κB and inflammasome pathways or inducing cytotoxic Ca^2+^ influx. Thus, the inflammatory milieu characteristic of GN delivers a set of shared signals capable of igniting several cell death pathways simultaneously.

### 4.2 Inflammation as a linking hub

Inflammation, particularly the sterile inflammation provoked by urate crystals, serves as a central hub that links various PCD modalities in GN. Many of the cell death processes either drive inflammation or are themselves driven by it, creating a network of positive feedback that amplifies renal injury.

IL-1β and TNF-α act as critical node connectors within this network (18). IL−1β, primarily released during pyroptosis, can amplify other PCD pathways. It recruits neutrophils that release proteases and ROS, which in turn cause bystander apoptosis or necroptosis in tubular cells. IL−1β also upregulates adhesion molecules on endothelial cells, promoting microvascular inflammation and local ischemia, thereby triggering further tubular apoptosis (117). TNF−α, often produced by macrophages in GN, can directly induce apoptotic or necroptotic death of tubular cells. Together, IL−1β and TNF−α establish a vicious cycle. MSU crystals stimulate their release through pyroptosis and necroptosis, and these cytokines subsequently kill more cells or prime them for inflammasome activation, leading to yet more IL−1β production (18).

The NLRP3 inflammasome occupies a pivotal position at the crossroads of inflammation and cell death. While its activation leads to pyroptosis, NLRP3 also exerts non−pyroptotic effects (118). In renal cells, NLRP3 activation initiates an inflammatory cascade that heightens susceptibility to apoptosis or necroptosis (118). For example, IL−1 released upon NLRP3 activation can downregulate survival factors and, through IL−1 receptor signaling, reduce intracellular K^+^ levels, potentially destabilizing cell membranes. Conversely, signals derived from apoptosis, such as fragmented DNA, and from necroptosis, such as released HMGB1, can further activate NLRP3 in neighboring cells (5). Thus, NLRP3 is both a recipient and sender of inter-pathway signals, anchoring a network where death begets inflammation, which begets more death.

Necroinflammation, a concept describing the inflammatory response triggered by regulated necrotic forms of cell death such as necroptosis, pyroptosis, and ferroptosis, further reinforces this interconnectivity (119). In GN, cells dying via these pathways release DAMPs that engage pattern−recognition receptors on surviving cells and infiltrating leukocytes, frequently activating NF−κB and inducing pro−inflammatory gene expression (9). This creates a signaling landscape where NF−κB serves as a common convergence point, activated by TNF from necroptosis, IL−1 from pyroptosis, or TLRs sensing HMGB1 from any necrotic death. In turn, NF−κB can upregulate death ligands such as Fas ligand or TNF, promoting apoptosis, or prime additional inflammasomes, fueling pyroptosis. In later fibrotic stages of the disease, necroinflammation also sustains chronic macrophage activation and T−cell recruitment, maintaining epithelial cells in a sub−lethal stressed state that remains prone to PCD.

A key feature of this integrated PCD and inflammation network is its capacity for feed−forward amplification. A relatively small initial insult, such as a few crystals inducing pyroptosis in a limited number of macrophages, can through inflammatory amplification result in widespread injury marked by extensive pyroptosis and necroptosis in tubular epithelium (9). Each dying cell can incite the demise of its neighbors, a phenomenon reminiscent of a bystander effect (120). For instance, in hyperuricemic mice, genetic deletion of RIPK3 not only reduced necroptosis but also lowered IL−1β levels and caspase−1 activation (5), implying that RIPK3−mediated cell death normally fuels inflammasome activity, likely via DAMPs that sustain macrophage and NLRP3 activation. Similarly, suppressing NLRP3 not only diminishes pyroptosis but can indirectly reduce necroptosis and apoptosis by lowering the local availability of IL−1 and TNF (5, 11).

### 4.3 Oxidative stress and organelle damage

Dysfunction of key organelles, especially mitochondria and lysosomes, under oxidative stress represents a central event that integrates the web of PCD in GN (9, 121, 122). These organelles serve as platforms where signals for distinct death pathways both originate and intersect.

Mitochondria play multiple, converging roles in this network. They act not only as executioners of apoptosis through cytochrome c release and caspase−9 activation, but also as a major source of ROS that drive ferroptosis and inflammasome activation. In hyperuricemic conditions, mitochondrial DNA is susceptible to oxidative damage, and its release into the cytosol functions as a DAMP that directly activates the NLRP3 inflammasome, thereby linking mitochondrial injury to pyroptosis (22). Mitochondrial ROS also lower the threshold for the mitochondrial permeability transition, promoting necrotic forms of death, or can trigger apoptosis by oxidizing cardiolipin and facilitating Bax insertion into the mitochondrial membrane (123, 124). Furthermore, mitochondrial dysfunction reduces ATP production, which impairs membrane ion pumps and disturbs ionic homeostasis, further exacerbating cell death. Notably, mitochondrial lipid peroxides are important triggers for ferroptosis; under physiological conditions, GPX4 repairs such damage, but hyperuricemia decreases GPX4 expression (76). Consequently, a single damaged mitochondrion in a urate−laden cell may simultaneously release signals that promote apoptosis, initiate pyroptosis via mitochondrial DNA, and trigger ferroptosis via lipid peroxides, positioning mitochondria as a central hub for PCD signaling (121).

Lysosomes also occupy a critical position, participating both in autophagy and as triggers for inflammasome−mediated pyroptosis. Their membrane integrity is compromised by phagocytosed crystals and by iron−catalyzed ROS, since lysosomes store iron and are vulnerable to peroxidation. Leakage of lysosomal contents, such as the acid hydrolase cathepsin B, can initiate apoptosis by cleaving Bid into its truncated form (125), thereby promoting mitochondrial outer membrane permeabilization, and can simultaneously drive inflammasome assembly via NLRP3 activation (20). Lysosomal rupture essentially marks a point of no return for cellular homeostasis, commonly leading to death through either apoptosis or pyroptosis. In the context of ferroptosis, lysosomes may accumulate iron, and under conditions of iron overload, their membranes become prone to oxidative rupture, which can concurrently activate NLRP3 and induce necrotic−like cell death (126). Thus, the lysosome functions as a convergence point where autophagic processing and inflammasome activation meet; excessive autophagic cargo, as often occurs in GN, can render lysosomes fragile, thereby converting a survival mechanism into a potent death trigger.

The endoplasmic reticulum and its calcium stores further contribute to this network (127). Uric acid can induce protein−misfolding stress within the endoplasmic reticulum, leading to the unfolded protein response (128). When this response is overwhelmed, it triggers CHOP (C/EBP homologous protein)-mediated apoptosis. As the primary intracellular calcium store, the endoplasmic reticulum can release Ca^2+^ into the cytosol under stress, promoting calpain activation and mitochondrial calcium overload, events that bridge to apoptosis and necroptosis (127). Endoplasmic reticulum stress has also been linked to the priming of the NLRP3 inflammasome (129).

The interplay among these organelles and the existence of threshold effects are fundamental to understanding the integrated death network. Cells possess adaptive responses, such as the unfolded protein response for endoplasmic reticulum stress, mitophagy for damaged mitochondria, and lysophagy for injured lysosomes. In GN, however, these adaptive mechanisms often prove insufficient. Autophagy represents one such compensatory response that attempts to manage organelle damage—for example, by removing ROS−generating mitochondria via mitophagy or clearing protein aggregates resulting from endoplasmic reticulum stress (130). Yet, when hyperuricemia continuously inflicts organelle injury, even autophagy can become overwhelmed or dysregulated, as we discussed. This overload ultimately reaches a tipping point at which organelle failure triggers catastrophic activation of multiple PCD pathways.

### 4.4 Cell type-specific interactions

The renal environment in GN comprises multiple cell types, including tubular epithelial cells, glomerular cells, interstitial fibroblasts, endothelial cells, and infiltrating immune cells such as macrophages and neutrophils. The PCD network operates differently in each of these cell populations, and their interactions play a crucial role in driving renal injury (9).

Tubular epithelial cells are the principal target of urate toxicity and may undergo apoptosis, pyroptosis, ferroptosis, and possibly necroptosis (16). When tubular cells die, they release DAMPs that activate interstitial macrophages. IL−1β produced by tubular cells during pyroptosis can also act on neighboring fibroblasts and endothelial cells, provoking local inflammatory and fibrotic responses (131). Under stress, tubular cells secrete cytokines such as MCP−1, which further recruit immune cells. A distinctive feature of tubular cells is their ability to persist for a period in a dysfunctional, sublethal state characterized by partial activation of PCD pathways—for example, with limited mitochondrial damage that does not immediately cause death. In this state, they may secrete pro−fibrotic factors, resembling a senescence−associated secretory phenotype (SASP), thereby promoting fibrosis even before full cell demise (132, 133).

Macrophages function both as victims and perpetuators of PCD processes. Upon phagocytosing MSU crystals, macrophages readily undergo pyroptosis, releasing IL−1β that affects other cells (11, 61). In a high-TNF−α and crystal−rich environment, macrophages can also undergo necroptosis, which leads to the release of cellular contents and escalation of inflammation (134). Additionally, macrophages phagocytose debris from apoptotic tubular cells; when overloaded, this can drive macrophages into a pro−inflammatory state, similar to lipid−overloaded macrophages in atherosclerosis (135). There is a reciprocal interplay in which tubular cells undergoing necroptosis release signals that recruit macrophages, and those macrophages, if they subsequently die via pyroptosis or necroptosis, generate a toxic milieu that further damages tubular cells—creating a destructive dialogue between epithelial and immune compartments.

Although less studied in chronic GN, neutrophils contribute to acute renal injury through the formation of neutrophil extracellular traps (NETs) (136). NETosis, a distinct form of cell death, involves the extrusion of chromatin to entrap crystals, which can also inflict tissue damage. Within the kidney, NETs may occlude capillaries or directly injure endothelial cells. Components of NETs, such as histones, are highly cytotoxic and pro−thrombotic, potentially exacerbating renal injury. Moreover, IL−1β derived from pyroptosis can enhance NET formation, and conversely, NETs may stimulate inflammasome activation in macrophages. Thus, neutrophils add complexity to the PCD network via NETosis and potentially through secondary necrosis.

Fibroblasts and myofibroblasts act as end−stage effector cells in CKD, responsible for collagen deposition and scar formation (137). They are often activated by TGF−β, which can be released from apoptotic bodies of tubular cells or secreted by macrophages (138). While fibroblasts themselves exhibit relative resistance to many forms of PCD, under severe oxidative or inflammatory stress they may undergo apoptosis, which can partially limit fibrogenesis. However, ongoing necroinflammation continuously recruits and activates new fibroblasts. Furthermore, apoptosis of endothelial cells, leading to capillary rarefaction, indirectly stimulates fibroblasts through hypoxia−dependent mechanisms. Consequently, cell death in one compartment, such as the microvasculature, can drive fibrotic responses in another, such as the interstitium.

Endothelial cells are also vulnerable to hyperuricemic damage. Studies indicate that high urate levels can induce endothelial pyroptosis via ROS and NLRP3 inflammasome activation (139). Endothelial cell death reduces tissue perfusion, contributing to ischemic tubular injury and thereby linking endothelial PCD to tubular PCD. Additionally, endothelial cells express adhesion molecules that facilitate immune cell infiltration. When endothelial cells undergo PCD, they may expose pro−coagulant surfaces, promoting microthrombus formation and leading to local infarcts and necrosis, which further amplifies renal injury.

## 5 Therapeutic implications of targeting PCD in GN

### 5.1 Mitigating apoptosis and oxidative stress

Current treatment of GN largely revolves around lowering serum urate levels, typically using xanthine oxidase inhibitors such as allopurinol or febuxostat (6). While this approach addresses the fundamental metabolic disturbance, it does not directly counteract the cellular damage already established within the kidneys. Targeting apoptosis and the oxidative stress that triggers it may offer renal protection that extends beyond mere urate reduction.

Antioxidant therapy is a logical strategy. By scavenging ROS or inhibiting their production, it is possible to interrupt the pathogenic cascade that progresses from urate accumulation to ROS generation, mitochondrial damage, and ultimately apoptosis. For instance, inhibitors of NADPH oxidase could be repurposed for this purpose. Studies have demonstrated that diphenyleneiodonium (DPI), a NADPH oxidase inhibitor, prevented ROS generation and significantly reduced apoptosis in tubular cells exposed to uric acid. Similarly, knockdown of NOX4 blocked the apoptotic cascade in these cells (27). These findings suggest that pharmacological agents such as apocynin or other NOX inhibitors might attenuate tubular apoptosis in GN.

Another promising approach involves preserving mitochondrial integrity. Mitochondria−targeted antioxidants, including compounds like MitoQ or the SS−31 peptide, can neutralize ROS at their source and help stabilize mitochondrial membranes, thereby preventing the release of cytochrome c and subsequent activation of the intrinsic apoptotic pathway (140, 141). Modulating the balance between pro− and anti−apoptotic Bcl−2 family proteins, for example by using Bcl−2 mimetics or up−regulators, could favor cell survival under stress. Conversely, inhibiting excessive p53 activation through p53 inhibitors or perhaps using sirtuin activators that deacetylate p53 may reduce the intrinsic apoptotic drive induced by hyperuricemia.

Preventing urate entry into tubular epithelial cells offers a way to intercept the problem at its origin. URAT1 inhibitors, including uricosuric drugs such as probenecid and lesinurad, not only lower serum urate but also protect tubular cells by reducing intracellular urate accumulation. In cell culture, probenecid or knocking down URAT1 markedly blunted uric acid–induced apoptosis (27). Losartan, an angiotensin receptor blocker with mild uricosuric effect, similarly decreased tubular apoptosis in hyperuricemic models, indicating dual benefits of lowering intraglomerular pressure and urate reabsorption.

Emerging therapeutic avenues also aim at the nexus of metabolism and oxidative stress. Xanthine oxidase inhibitors themselves can reduce ROS production, given that xanthine oxidase generates superoxide as a by−product during uric acid synthesis (142). In this regard, allopurinol and febuxostat may provide renal benefit not only by lowering serum urate but also by decreasing xanthine oxidoreductase-derived oxidative stress. Febuxostat has been reported to attenuate ER stress-mediated tubular injury and apoptosis in experimental hyperuricemic nephropathy, suggesting a potential link between conventional urate-lowering therapy and modulation of apoptosis-related cellular stress pathways (143). Nutraceutical antioxidants, such as vitamin C, curcumin, or quercetin, may provide additional support. Quercetin, in particular, exhibits both urate−lowering and anti−apoptotic properties, likely mediated through activation of NRF2 and heme oxygenase−1 (144). In hyperuricemic animal models, quercetin administration reduced renal apoptosis and improved renal function, probably by enhancing antioxidant defenses and suppressing inflammation (39).

### 5.2 Blocking necroptosis to halt tissue necrosis and inflammation

Given the evidence that necroptosis plays a pivotal role in crystal−induced kidney damage (5, 15), inhibiting this pathway offers a way to reduce both cell loss and inflammatory signaling in GN. The necroptosis cascade presents several druggable nodes that could be targeted therapeutically.

Inhibition of RIPK1 represents one such approach. Necrostatin−1, a prototype RIPK1 kinase inhibitor, has been shown to block necroptosis across various preclinical models and effectively prevented monosodium urate−induced cell death in studies of crystal cytotoxicity (15). Newer, more specific RIPK1 inhibitors, such as GSK2982772—currently entering clinical trials for inflammatory diseases—could also be considered for hyperuricemia−related kidney injury. These agents might not only suppress necroptotic death of tubular cells but also attenuate the secondary inflammation triggered by such death. It should be noted, however, that RIPK1 participates in other cellular processes, including apoptosis via complex IIa with FADD and NF-κB activation. While systemic RIPK1 inhibition may therefore have complex effects, in renal contexts the net outcome appears protective in models of both acute and chronic kidney injury (145).

Targeting downstream components of the pathway, such as RIPK3 and MLKL, offers alternative or complementary strategies. Although no RIPK3 inhibitors are yet clinically available, experimental compounds like GSK’872 can inhibit its kinase activity. A more direct intervention involves blocking MLKL, the terminal executor of necroptosis. Necrosulfonamide, an inhibitor of human MLKL, suppressed crystal−induced cell death in human renal cells *in vitro* (15). If a safe, systemically active MLKL inhibitor could be developed, it would provide a means to specifically prevent cell membrane rupture without broadly interfering with upstream TNF signaling, an advantage over more upstream inhibitors like those targeting RIPK1.

Since TNF-α is a major driver of necroptosis in inflammatory environments, the use of TNF−α inhibitors such as etanercept or infliximab might indirectly reduce necroptotic tubular injury. These systemic immunosuppressive agents have not been studied specifically in GN, but in patients with severe gout and systemic inflammation, they could potentially offer dual benefits by alleviating both articular and renal damage. It is important to recognize, however, that TNF−α also plays certain protective roles, including aiding crystal clearance through granuloma formation, so careful patient selection would be essential.

Combining necroptosis inhibitors with anti−inflammatory agents may yield synergistic effects. For instance, blocking necroptosis reduces the release of DAMPs such as HMGB1, which normally primes inflammasomes, thereby lowering the inflammatory burden. Conversely, inhibiting IL−1 might raise the threshold for cells to undergo necroptosis by reducing extrinsic stress. Thus, a combination of Necrostatin−1 with an IL−1 blocker like anakinra could theoretically provide greater renal protection than either agent alone, though this hypothesis remains speculative and requires experimental validation.

### 5.3 Inhibiting pyroptosis and inflammasome activation

Given the central role of the NLRP3 inflammasome and IL−1β in gout−associated inflammation, therapeutic strategies targeting pyroptosis are highly relevant to GN. Indeed, gout was among the first diseases in which IL−1 blockade proved dramatically effective in articular inflammation, and similar principles can be applied to protect the kidney (146).

Inhibitors of the NLRP3 inflammasome, such as the compound MCC950 (also known as CRID3), specifically block NLRP3 activation (147). MCC950 has demonstrated efficacy across various inflammatory disease models and, under hyperuricemic conditions, would be expected to prevent caspase−1 activation and subsequent cleavage of GSDMD, thereby halting pyroptosis at an early stage. Although not yet approved for clinical use, such inhibitors are under development for conditions like cryopyrin−associated periodic syndromes and could be repurposed for gout−related inflammation. Natural compounds, including certain flavonoids, also exhibit partial NLRP3−inhibitory activity.

Among clinically used urate-lowering agents, febuxostat has attracted particular attention because it may interfere with inflammasome signaling beyond urate reduction. Experimental studies have shown that febuxostat suppresses NLRP3 inflammasome-mediated IL-1β secretion and inflammatory cell death through both mitochondrial ROS-dependent and mitochondrial ROS-independent mechanisms, the latter involving improved intracellular ATP availability and mitochondrial energetics (148). These findings suggest that febuxostat may bridge conventional urate-lowering therapy and PCD-oriented intervention, especially in inflammasome-driven renal inflammation.

Direct inhibition of caspase−1 is another viable approach. Agents like VX−765, a prodrug of a caspase−1 inhibitor, can reduce the maturation of IL−1β and limit pyroptotic cell death. VX−765 has been evaluated in clinical trials for epilepsy and inflammatory disorders and, in principle, could diminish the release of IL−1β and IL−18 in GN, protecting renal cells from lytic death. While non−canonical pyroptosis mediated by caspase−11 (in rodents) or caspases−4/5 (in humans) offers an additional target, the canonical NLRP3−caspase−1 pathway is considered dominant in sterile urate−induced kidney injury.

Biologic agents that neutralize the downstream effectors of pyroptosis, such as the IL−1 receptor antagonist anakinra, the anti−IL−1β monoclonal antibody canakinumab, or IL−18−binding protein, offer a complementary strategy (146). In practice, IL−1 blockers are already used off−label for refractory gout flares. For patients with GN, these agents could reduce interstitial inflammation and disrupt the cycle of leukocyte recruitment and further cell death. Short courses of anakinra during acute exacerbations or in patients with high IL−1 burden might help preserve renal function. Although IL−18 is less frequently targeted clinically, its contribution to Th1 responses and fibrosis suggests that IL−18−binding protein could serve as a niche therapy if IL−18 is identified as a major driver in individual cases.

Targeting the final executioner of pyroptosis, GSDMD, provides a means to prevent membrane pore formation. The drug disulfiram, traditionally used in the management of alcoholism, was recently found to covalently modify GSDMD and inhibit pyroptosis (149). *In vitro*, disulfiram blocked GSDMD pore formation and IL−1β release triggered by inflammasome activation, and in a model of gouty arthritis, it reduced inflammation, indicating potential applicability in GN. It should be noted, however, that some studies suggest GSDMD−mediated pores may not be absolutely required for IL−1β release in all contexts of MSU stimulation, implying that crystals can elicit partial IL−1β secretion through alternative mechanisms. Thus, while GSDMD inhibitors can mitigate cell lysis and associated damage, upstream blockade of the NLRP3 inflammasome or caspase−1 may be more effective in comprehensively suppressing inflammation.

Colchicine, a traditional mainstay of gout therapy, also merits consideration for its ability to dampen pyroptosis. By inhibiting microtubule polymerization, colchicine interferes with both inflammasome assembly and the vesicular secretion of IL−1β. It has been shown to reduce NLRP3 inflammasome activity and IL−1 production by preventing the co−localization of inflammasome components. In the context of GN, colchicine could attenuate renal inflammation by suppressing pyroptosis in infiltrating macrophages. Additionally, it inhibits neutrophil chemotaxis, thereby reducing NETosis and neutrophil−mediated injury. Low−dose colchicine is relatively safe in patients with CKD, making it a feasible adjunctive therapy to curb crystal−driven inflammation in the kidney.

### 5.4 Targeting ferroptosis and disordered iron metabolism

Preventing ferroptotic cell death in GN is an appealing strategy to protect renal tubular epithelium from the synergistic damage caused by iron dysregulation and oxidative stress, which often operates in concert with other forms of PCD. Several approaches can be envisioned to intervene in this pathway.

Inhibiting lipid peroxidation stands as a direct and effective means to halt ferroptosis (150). Small−molecule radical−trapping antioxidants such as ferrostatin−1 and liproxstatin−1 specifically interrupt the chain reactions of lipid peroxidation. Studies have demonstrated that ferrostatin−1 significantly improved renal outcomes in hyperuricemic mouse models (36, 76). Although these compounds remain investigational, their mechanism parallels that of well−tolerated agents like vitamin E or tempol, which integrate into cell membranes and quench peroxyl radicals. High−dose vitamin E has exhibited renoprotective effects in certain CKD models by reducing lipid peroxide levels, suggesting it could likewise attenuate ferroptosis under hyperuricemic conditions (151).

Iron chelation offers another rational approach by removing the catalytic iron that fuels the Fenton reaction and drives lipid peroxidation. Clinically available iron chelators, including the injectable agent deferoxamine and oral drugs such as deferiprone and deferasirox, could be considered if renal iron overload is documented. In hyperuricemic models, testing whether an iron−restricted diet or chelation therapy lowers kidney injury markers would be informative. Chelators might also modestly influence crystal formation, as iron can catalyze the oxidation of urate to less soluble forms. Caution is warranted, however, because iron is essential for normal cellular functions; therefore, any chelation regimen would require careful dosing to avoid inducing anemia or other deficiencies.

Activating the NRF2 pathway can enhance the expression of a network of antioxidant and iron−handling proteins, including heme oxygenase−1, ferritin, GPX4, and the cystine transporter xCT. Although the NRF2 activator bardoxolone methyl was discontinued in CKD trials due to safety concerns (152), the concept of boosting NRF2 signaling to mitigate oxidative renal damage remains valid (153). In GN, milder NRF2 activators or targeted delivery systems might strengthen cellular defenses against ferroptosis. Natural compounds such as curcumin, sulforaphane (derived from cruciferous vegetables), or bardoxolone analogs could potentially upregulate NRF2 and subsequently increase GPX4 levels, thereby restraining lipid peroxidation. As noted earlier, pachymic acid has been reported to directly bind and activate NRF2, resulting in reduced ferroptosis and fibrosis in hyperuricemic models (78).

Supplementing glutathione precursors addresses the depletion of glutathione that precipitates ferroptosis. Providing building blocks such as N−acetylcysteine can elevate intracellular glutathione levels and help sustain GPX4 activity. N−acetylcysteine is a well−tolerated drug already repurposed to mitigate contrast−induced nephropathy by enhancing cellular redox capacity (154, 155). By maintaining high glutathione concentrations, N−acetylcysteine or similar thiol donors might slow the progression of ferroptotic injury in hyperuricemic kidneys.

### 5.5 Autophagy modulation: timing and context–dependent interventions

Given the dual nature of autophagy in GN—exhibiting both protective and pathogenic effects—therapeutic modulation of this process requires a nuanced and context−sensitive approach. The overarching goal is to restore appropriate autophagic flux: sufficient to clear harmful cellular debris and damaged organelles, yet not so excessive as to provoke self−injury or amplify inflammasome−driven inflammation (156, 157). One potential molecular switch underlying this transition is the Beclin-1/Bcl-2 regulatory axis: Bcl-2 restrains Beclin-1-dependent autophagy under basal conditions, whereas sustained cellular stress may disrupt this interaction and promote Beclin-1/Vps34-driven autophagosome formation (158). In GN, chronic urate exposure, ROS accumulation, and ER stress may therefore shift autophagy from an adaptive clearance response toward excessive or maladaptive activation when the stress is prolonged.

In situations where autophagy is pathologically overactivated, inhibition may be beneficial. The study by Hu et al. demonstrated that 3−methyladenine, an autophagy inhibitor, alleviated hyperuricemic nephropathy by reducing NLRP3−dependent pyroptosis (20). This suggests that under conditions of sustained hyperuricemia and persistent crystal stress, temporarily restraining autophagy can diminish the release of cathepsin B and attenuate inflammatory injury (159). This pathogenic phase may reflect exhaustion of lysosomal degradative capacity under chronic urate load: soluble urate and MSU crystals continuously increase the burden of damaged mitochondria, oxidized proteins, and crystal-containing vesicles that must be processed through the autophagy–lysosome system. Once lysosomal clearance becomes insufficient or lysosomal membranes are destabilized, cathepsin B leakage may activate NLRP3 inflammasome signaling and convert initially protective autophagy into an amplifier of pyroptosis and inflammation. Pharmacological interventions could include research−grade agents such as 3−methyladenine or inhibitors targeting key autophagy regulators like Beclin−1 or Vps34 (159). Hydroxychloroquine, a lysosomotropic drug that blocks autophagosome−lysosome fusion and is already used in lupus nephritis, might also be repurposed. By raising lysosomal pH, hydroxychloroquine could impair the degradation of autophagic cargo and potentially reduce cathepsin leakage. Caution is warranted, however, because such an intervention might simultaneously hinder the clearance of monosodium urate crystals, making its net effect in GN uncertain.

Conversely, in acute settings such as a renal gout flare or early hyperuricemic stress, enhancing autophagy might help cells cope with the sudden insult. Mammalian target of rapamycin (mTOR) inhibitors, including rapamycin, induce autophagy and have shown protective effects in models of acute kidney injury by promoting the removal of damaged mitochondria (160). In theory, rapamycin could assist tubular epithelial cells in processing an abrupt load of crystals or oxidized material, thereby limiting cell death (161, 162). Moreover, rapamycin−induced autophagy can degrade components of the inflammasome pathway, such as pro−IL−1β and NLRP3, leading to reduced IL-1β release (100). In gouty arthritis, rapamycin analogs have been discussed as a means to promote crystal sequestration and dampen inflammation, raising the possibility of similar benefits in renal involvement.

The timing of intervention, often referred to as the therapeutic window, is a critical consideration. Early in the course of GN, before extensive tubular loss has occurred, autophagy enhancers might prolong cellular survival. In later stages, where autophagy may become hyperactivated and contribute to sustained inflammation, inhibitors could be more appropriate. It is conceivable that an individual patient might benefit from autophagy induction at one phase of the disease and from inhibition at another. Currently, however, we lack reliable real−time clinical markers of renal autophagic activity. The analysis of urinary biomarkers, such as p62 or fragments of microtubule−associated protein 1A/1B−light chain 3 (LC3), might offer indirect clues to autophagic flux in the kidney.

A major challenge in PCD-targeted therapy is biological redundancy and pathway switching. Inhibition of one execution pathway may not necessarily restore cell survival if upstream urate-induced stress persists. For example, blockade of the NLRP3/caspase-1/GSDMD axis may reduce pyroptotic pore formation and IL-1β release, but residual MSU crystal-induced lysosomal injury, ROS accumulation and TNF-α-rich inflammatory priming may still drive alternative forms of regulated death, including RIPK3–MLKL-mediated necroptosis or caspase-dependent apoptosis. This concept is supported by broader evidence that pyroptosis, apoptosis and necroptosis are extensively interconnected and may compensate for one another under inflammatory stress. Therefore, therapeutic strategies in GN may need to move beyond single-node blockade toward context-dependent multi-node modulation, combining urate lowering, suppression of inflammasome activation, mitochondrial/oxidative stress control and selective inhibition of necroptotic or ferroptotic execution when appropriate. However, indiscriminate inhibition of multiple PCD pathways should be avoided, as physiological cell death is essential for immune defense, tissue remodeling and resolution of inflammation.

## 6 Conclusions and perspectives

Long regarded as a metabolic stone disease, GN is now understood to be driven by a self−sustaining network of PCD and sterile inflammation (163). Elevated uric acid and urate crystals initiate a complex interplay of apoptosis, necroptosis, pyroptosis, ferroptosis, and autophagy within the kidney. Together, these processes lead to tubular injury, interstitial inflammation, and fibrotic remodeling. Rather than operating in isolation, these PCD pathways form an integrated “death web” that shares common triggers such as oxidative stress and crystal−induced damage and amplifies itself through inflammatory mediators and organelle dysfunction. This network perspective clarifies why simply lowering uric acid may not fully arrest kidney disease progression in gout: once the cycle of cell death and inflammation is established, it can maintain tissue injury even when systemic urate levels are reduced (164, 165).

Autophagy emerges as a critical regulator within this network, capable of both attenuating and aggravating the damage. It acts as the cell’s internal rheostat for stress, attempting to modulate the effects of urate in GN but often becoming dysregulated in the process. Consequently, autophagy represents a potential therapeutic node; by modulating its activity, we can indirectly influence multiple forms of cell death—for example, by reducing inflammasome activation or preserving mitochondrial integrity. However, its dual−edged nature demands carefully calibrated interventions.

Looking forward, therapies that target the PCD network in GN promise to transform clinical management (**Table 2**). We anticipate a shift from a traditionally uric acid−centric approach toward a strategy centered on cellular protection and inflammation resolution. Such an approach could combine a xanthine oxidase inhibitor to lower the urate burden, an IL−1β blocker to dampen inflammation and pyroptosis (146, 166), a small−molecule inhibitor of RIPK1 or ferroptosis to limit ongoing necrotic cell loss (167), and possibly an agent that normalizes autophagic flux. While these combination regimens require rigorous testing for safety and additive efficacy, the rationale for simultaneously addressing multiple arms of the death network is compelling and likely to yield synergistic benefits.

**Table 2:** Representative pharmacological agents targeting PCD-related pathways in GN.

PCD pathway	Representative agent	Main target/mechanism	Stage/evidence
Apoptosis/oxidative stress	Febuxostat	XO inhibition; ROS and ER stress reduction	Clinical drug; experimental evidence in hyperuricemic nephropathy
	Quercetin	NRF2/HO-1 activation; anti-oxidative and anti-apoptotic effects	Preclinical evidence in hyperuricemic models
	MitoQ; SS-31	Mitochondrial ROS/injury	Preclinical/clinical exploration
Necroptosis	Necrostatin-1/Nec-1s	RIPK1 inhibition	Preclinical
	GSK2982772	RIPK1 inhibition	Clinical trial for inflammatory diseases; not tested in GN
Pyroptosis	MCC950 (171)	NLRP3 inhibition	Preclinical; tested in crystal nephropathy models
	Dapansutrile/OLT1177	NLRP3 inhibition	Clinical trial for gout flares; not tested in GN
	VX-765	Caspase-1 inhibition	Clinical trial; not tested in GN
Ferroptosis	Ferrostatin-1 (36)	Lipid peroxidation inhibition	Preclinical evidence in hyperuricemic nephropathy
	Liproxstatin-1	Lipid peroxidation inhibition	Preclinical
	Pachymic acid	NRF2 activation; ferroptosis suppression	Preclinical evidence in hyperuricemic renal injury
Autophagy	3-Methyladenine (20)	PI3K/Vps34-related autophagy inhibition	Preclinical evidence in hyperuricemic nephropathy
	Rapamycin	mTOR inhibition; autophagy induction	Approved for other indications; experimental concept in GN

However, translating this therapeutic concept into clinical practice will require careful interpretation of the experimental evidence on which it is based. Much of our current understanding of PCD in GN comes from rodent models of experimentally induced hyperuricemia, particularly potassium oxonate-based protocols that inhibit uricase and rapidly elevate serum urate. These models are useful for establishing causal links between urate overload, oxidative stress, inflammation, tubular injury and PCD activation, but they inevitably compress into days or weeks a disease process that in humans usually develops over years to decades. This temporal mismatch is especially important because human GN evolves under fluctuating urate exposure, recurrent or persistent MSU crystal deposition, aging, metabolic comorbidities and long-term renal remodeling. Moreover, most rodents naturally express uricase, whereas humans lack functional uricase; thus, pharmacological uricase inhibition creates a useful but artificial approximation of human hyperuricemia. Current model reviews similarly emphasize that chemically induced hyperuricemia models are indispensable for mechanistic discovery but differ from human disease in urate physiology, model stability, crystal deposition patterns and chronicity. Therefore, PCD-targeted interventions that appear effective in rapid-onset animal models should be validated in more chronic and human-relevant systems before being translated to patients with GN. Future studies should incorporate uricase-deficient or humanized urate-handling models, aged animals with metabolic comorbidities, longitudinal sampling, patient-derived renal tissue, urinary extracellular vesicles, organoids and spatial or single-cell analyses to better define stage- and cell-type-specific PCD signatures in human disease.

Translational research should prioritize the identification of biomarkers that reflect active PCD pathways in patients. For instance, urinary IL-18 may signal inflammasome activation (168), urinary neutrophil gelatinase−associated lipocalin might indicate tubular epithelial stress linked to apoptosis (169), and specific lipid peroxidation products could serve as indicators of ferroptosis. Such markers could help stratify patients and guide the selection of targeted therapies. There is also growing interest in whether early intervention could entirely prevent GN in hyperuricemic patients. If subclinical tubular cell death could be detected at its onset, for example, through a subtle rise in kidney injury molecule−1 (KIM-1) (170), short−term administration of antioxidants or IL−1 inhibitors might be used preemptively to disrupt the initiating cycle of PCD.

In summary, the paradigm of GN is evolving from a simple depositional disorder to a complex immuno−nephrological condition in which PCD serves as the pivotal mechanism determining renal survival or destruction. By deciphering and manipulating this network of PCD pathways, we stand at the threshold of novel therapies that could protect the kidneys of patients with gout, thereby slowing the progression of CKD and potentially improving long−term outcomes. The insights gained from studying GN may also illuminate other crystal−induced and metabolic kidney diseases, establishing it as a model for understanding how chronic metabolic stress provokes organ damage through PCD and inflammation. With continued interdisciplinary research, the long−neglected “gouty kidney” may soon benefit from effective, targeted treatments that address a significant unmet need in nephrology.
